# Noradrenergic Modulation of the Piriform Cortex: A Possible Avenue for Understanding Pre-Clinical Alzheimer’s Disease Pathogenesis

**DOI:** 10.3389/fncel.2022.908758

**Published:** 2022-05-26

**Authors:** Vishaal Rajani, Qi Yuan

**Affiliations:** Division of Biomedical Sciences, Faculty of Medicine, Memorial University of Newfoundland, St. John’s, NL, Canada

**Keywords:** norepinephrine, piriform cortex, olfactory dysfunction, locus coeruleus, Alzheimer’s disease, L-type calcium channel

## Abstract

Olfactory dysfunction is one of the biomarkers for Alzheimer’s disease (AD) diagnosis and progression. Deficits with odor identification and discrimination are common symptoms of pre-clinical AD, preceding severe memory disorder observed in advanced stages. As a result, understanding mechanisms of olfactory impairment is a major focus in both human studies and animal models of AD. Pretangle tau, a precursor to tau tangles, is first observed in the locus coeruleus (LC). In a recent animal model, LC pretangle tau leads to LC fiber degeneration in the piriform cortex (PC), a cortical area associated with olfactory dysfunction in both human AD and rodent models. Here, we review the role of LC-sourced NE in modulation of PC activity and suggest mechanisms by which pretangle tau-mediated LC dysfunction may impact olfactory processing in preclinical stage of AD. Understanding mechanisms of early olfactory impairment in AD may provide a critical window for detection and intervention of disease progression.

## Introduction

Olfactory dysfunction is one of the earliest signs of Alzheimer’s Disease (AD), appearing long before clinical memory symptoms, at which point the disease has already progressed to late stages when intervention is difficult (Crous-Bou et al., 2017; Yan et al., 2022). Longitudinal studies demonstrate that early odor deficits predict the subsequent rate of episodic memory decline, and that worsening of these deficits is indicative of AD (Wilson et al., 2009). AD patients experience deficiencies in odor detection, identification and recognition memory (Mesholam et al., 1998). As a result, olfactory impairment has been adopted as a biomarker for pre-clinical AD, indicative of the presence of abnormal amyloid-beta (Aβ) and hyperphosphorylated tau proteins—hallmarks of disease onset—in otherwise cognitively healthy individuals (Xydakis and Belluscio, 2017; Murphy, 2019).

Impairment at all levels of the olfactory circuit have been implicated in AD development, from olfactory epithelium, nerve, olfactory bulb to olfactory cortex (Reyes et al., 1993; Kovacs et al., 1999; Arnold et al., 2010; Attems et al., 2015; Devanand, 2016; Bathini et al., 2019). Import to note, modified activity of the piriform cortex (PC), the primary olfactory cortex central to olfactory information encoding, is heavily implicated. Functional magnetic resonance imaging (fMRI), positron emission tomography (PET), and diffusion tensor imaging in patients with mild cognitive impairment (MCI) and AD symptoms show reduced PC activation during odor identification and perception tasks (Kareken et al., 2001; Li et al., 2010; Ebadi et al., 2017; Vasavada et al., 2017; Kjelvik et al., 2020; Donoshita et al., 2021). Moreover, pathological lesions observed exclusively within the PC correlate with impairment of odor identification and discrimination suggesting that this region plays a critical role in early olfactory deficits (Li et al., 2010; Kjelvik et al., 2020). In addition, both amyloid and tau generated animal models of AD exhibit neurodegeneration in olfactory regions and olfactory deficits (Cassano et al., 2011; Wesson et al., 2011; Saiz-Sanchez et al., 2012; Gandy et al., 2013; Kong et al., 2018; Adlimoghaddam et al., 2019; Ghosh et al., 2019). Several of these models point to PC specific dysfunction, due to increased expression of Aβ, tau and glycogen synthase kinase-3β in the PC compared to other odor sensory regions (Macknin et al., 2004; Cassano et al., 2011; Gandy et al., 2013), and direct disruption of PC function and PC-olfactory bulb (OB) coupling (Martinez-Garcia et al., 2021).

Recently, we presented novel results from a pretangle tau rat model and a new view that dysfunction of norepinephrine (NE) regulation in the PC may underlie preclinical stage olfactory deficiency (Ghosh et al., 2019; Omoluabi et al., 2021). In the rat pre-tangle tau model, pseudophosphorylated human tau is seeded in the NE-producing locus coeruleus (LC), mimicking the human origin of tau abnormality in the LC at young ages (Braak et al., 2011; Braak and Del Trecidi, 2015). LC pre-tangle tau, in the absence of amyloid plaques and neurofibrillary tangles, leads to degeneration of LC input to the PC, correlating with impaired olfactory discrimination learning (Ghosh et al., 2019). The degree of LC fiber degeneration within this model is inversely correlated with olfactory learning performance and preventing this degeneration in the PC rescued olfactory learning deficiency (Omoluabi et al., 2021). Together, LC degeneration is both sufficient and necessary for the olfactory discrimination deficiency observed in this rat model, consistent with a critical role of LC-NE input in the PC in various olfactory functions and learning (Doucette et al., 2007; Mandairon et al., 2008; Shakhawat et al., 2015). These findings recapitulate pre-clinical human AD conditions where both LC degeneration and olfactory dysfunction are predictive of AD progression (Chen et al., 2022; Yan et al., 2022). Altogether, this motivates us to dive into the mechanisms of noradrenergic modulation of cellular function and neural plasticity within the PC, with the hope of better understanding their roles in AD.

## Anatomy and Synaptic Plasticity of the Piriform Cortex

In mammals, the PC is central to olfactory information processing and is situated in the ventrolateral forebrain. It receives afferent input from the OB, rendering it as a critical site for odor discrimination, contextualization, and associative memory formation (Wilson and Sullivan, 2011; Bekkers and Suzuki, 2013; Blazing and Franks, 2020). Located parallel to the lateral olfactory tract (LOT), the PC extends from the anteriorly located OB to the lateral entorhinal cortex. In rodents, this rostro-caudal localization can be divided into distinct regions: the anterior (aPC) and posterior (pPC) piriform cortical regions, based on cell layer thickness and the location of the parallel LOT. Although they share similar input connections, the aPC and pPC vary in input distribution. This heterogeneity in anatomical structure may be important for the diverse roles of the PC in encoding sensory information (Wang et al., 2020).

**FIGURE 1:**
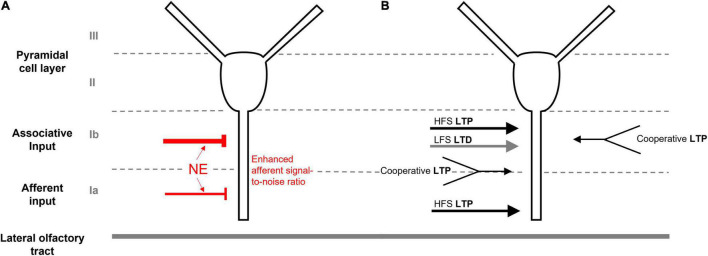
Anatomy and synaptic plasticity of the piriform cortex. **(A)** The unique laminar cytoarchitecture of the piriform cortex, consisting of cell body layers (II/III), the associative layer (Ib) and the afferent input layer (Ia). LC-sourced NE leads to stronger inhibition of associative connections in Ib and enhanced afferent signal-to noise ratio in Ia. **(B)** LTP can be induced by high frequence stimulation (HFS) of either the associative or afferent layers. Weak cooperative stimulation of either separate associative connections or co-activation of both afferent and associative fibers can also induce LTP in the presence of GABAA inhibition. Low frequence stimulation (LFS) can produce LTD in Ib.

At its core, the PC possesses a unique laminar cytoarchitecture that is amenable to the integration of afferent input with long lasting changes to synaptic strength (Figure 1A). Near the ventral surface, the LOT relays information from the OB via afferent synaptic connections (layer Ia) from cell body layers (layers II/III) consisting of pyramidal and semilunar cells. Adjacent to layer Ia, the layer of intrinsic associative connections (Ib) is indispensable for cortico-cortical signaling involved in associative memory formation. High frequency stimulation of either the associative or afferent layers in *ex vivo* slices can produce an *N*-methyl-D-aspartate receptor (NMDAR) dependent long-term potentiation (LTP) in pyramidal neurons, which is suggested to be essential for olfactory-based learning (Jung et al., 1990; Kanter and Haberly, 1990). LTP can also be produced by cooperative weak stimulation of separate associative fiber populations, or by co-activation of both afferent and intrinsic associative fibers in the presence of GABA_*A*_ inhibitors (Kanter and Haberly, 1993). With low frequency stimulation, long term depression (LTD) can also be induced and contributes to network stability following olfactory based learning (Lebel et al., 2001; Quinlan et al., 2004; Rajani et al., 2021 see Figure 1B for a summary of different types of synaptic plasticity). Afferent-mediated synaptic plasticity, however, seems limited to early developmental stages while associative plasticity persists in adult and aging rodents and may be modified by disease, aging, and learning states (Lebel et al., 2001; Poo and Isaacson, 2007; Rajani et al., 2021).

## Modulation of the Piriform Cortex by Norepinephrine

The LC is the main source of noradrenergic input to the PC, providing a homogeneous distribution of NE projections across anterior and posterior regions (Datiche and Cattarelli, 1996). Based on *in vitro* observations, released NE is suggested to activate inhibitory interneurons within the PC, producing inhibitory post-synaptic potentials (IPSPs) within the pyramidal cell layer (II) (Gellman and Aghajanian, 1993; Ghosh et al., 2015). NE signaling causes a higher suppression of associative layer (Ib) than afferent (Ia)-mediated excitatory postsynaptic potentials (EPSPs) of pyramidal neurons in *ex vivo* slices (Hasselmo et al., 1997). Computational models suggest that this dichotomous LC-mediated NE suppression enhances the signal-to-noise ratio of incoming afferent signals to increase odor learning (Hasselmo et al., 1997; de Almeida et al., 2015; Figure 1A).

Several rodent *in vivo* studies support the role of LC-NE in a diverse range of olfactory behaviors. Fitting with improving the signal-to-noise ratio, blocking α and β adrenergic receptors within the OB impaired similar odor discrimination in awake-behaving animals (Doucette et al., 2007), and modified the odor threshold required to perform reward-motivated odor tasks (Escanilla et al., 2010, 2012). Moreover, α_1_ and β receptors within the OB bidirectionally modulated discrimination of spontaneous odors and short-term odor habituation (Veyrac et al., 2007; Guerin et al., 2008; Mandairon et al., 2008; Shea et al., 2008). The utility of NE signaling extends to odor associative learning mediated within the PC. NE receptor activation within the PC is required for odor discrimination learning (Shakhawat et al., 2015), and via LC stimulation, enhanced PC neuronal responses to odor stimuli, sharpening odor representations (Bouret and Sara, 2002). More recently, optogenetics permitted the selective activation of LC noradrenergic neurons with specific activation patterns. Bilateral 10 Hz phasic stimulation of the LC enhanced similar odor discrimination learning in rats and this enhancement was occluded by a mixture of adrenergic receptor antagonists infused into the PC (Ghosh et al., 2021). Interestingly, learning facilitation was not produced by tonic LC stimulation at the same frequency, suggesting differential modes of LC release and subsequent uptake may influence the circuitries involved in learning as well as local adrenoceptor engagement. Furthermore, tonic and phasic LC activation generated opposite valence coding through the activation of the basolateral amygdala. High tonic stimulation (25 Hz) that was associated with a stress phenotype produced odor aversion, whereas the learning-promoting phasic stimulation (10 Hz) yielded odor preference (Ghosh et al., 2021; Omoluabi et al., 2022).

Another role for NE signaling within the PC is demonstrated in early odor preference learning, characterized by a heightened ability to form odor preference in neonatal rodents. In this early developmental learning model, NE signals via both α_2_ and β-adrenergic receptors at mitral cells within the OB to promote synaptic plasticity (Yuan et al., 2014). β-adrenergic receptor activation of PC pyramidal neurons is also essential, triggering an increase in cAMP response element binding protein (CREB) phosphorylation and enhancing theta-burst, NMDAR-mediated LTP induction at the LOT-aPC synapse (Morrison et al., 2013). This NE-mediated signaling seems locked to a specific developmental stage in rodents, enhancing miniature EPSC frequency at low doses in P8-11 mice, while increasing inhibition in mice after P14 (Ghosh et al., 2015). Downstream of β-adrenergic receptor activation, L-type calcium channel (LTCC) activity is enhanced via the cAMP/PKA pathway, facilitating CREB phosphorylation necessary for early odor preference learning (Mukherjee and Yuan, 2016; Ghosh et al., 2017b). Indeed, inhibition of PC LTCCs during early odor preference training in mice specifically impaired long-term odor memory (Mukherjee and Yuan, 2016).

This emerging evidence supports a critical role for LC-derived NE in multiple olfactory behaviors including odor detection, discrimination, and long-term associative learning. As a result, dysfunction of this neuromodulatory pathway produces measurable olfactory behavioral outcomes and is relevant to understanding disease pathogenesis.

## Norepinephrine and Olfactory Dysfunction in Humans and Animal Models

The LC, the main source of NE to the PC, is one of the key regions affected in early AD. Reduced LC volume and integrity, cell number and fiber density are closely correlated with cognitive decline and progressive AD stages (Chen et al., 2022). The LC is the initial site of expression of pretangle tau, a soluble, persistently phosphorylated precursor of neurofibrillary tangle formation, which spreads throughout the brain over the course of the human lifespan (Braak et al., 2011). LC pretangle tau first appears at young ages (childhood or puberty) (Braak and Del Tredici, 2011, 2012). By middle-age, pretangle tau is expressed within the entorhinal cortex, and later spreads into the hippocampus and cortical areas (Braak and Del Trecidi, 2015). On the other hand, the earliest tangles reported are associated with the anterior olfactory nucleus (a component of the prepiriform/piriform cortex), and entorhinal cortex, leading to synaptic plasticity impairment and cognitive decline (Price et al., 1991; Kovacs et al., 2001). This path of tau pretangle and tangle in the PC network and NE dysfunction in the PC due to LC pretangle tau could both critically influence early AD pathology, underlying impaired odor detection, discrimination and associative memory as observed in pre-clinical AD (Mesholam et al., 1998; Ghosh et al., 2019).

A decrease in LC-sourced NE has long been implicated in AD pathology, with reported neuronal loss within the LC and reduced NE inputs to other brain regions (Gulyas et al., 2010; Gannon et al., 2015; Theofilas et al., 2017). However, extracellular NE levels are not always decreased in AD patients, and adrenergic receptors have been reported to have a varied expression and density (Gannon and Wang, 2019). As a result, it is suggested that NE dysfunction within AD pathology may not be solely due to deficiency in NE inputs, but a part of a more complex system of changes at the receptor level involving neurodegenerative processes and compensatory mechanisms (Gannon and Wang, 2019). Given the fundamental importance of NE signaling to the enhancement of PC synaptic plasticity (Morrison et al., 2013; Ghosh et al., 2017a), odor valence and discrimination (Shakhawat et al., 2015; Ghosh et al., 2021), it is plausible that the early stages of soluble pretangle tau initially expressed within the LC could directly influence olfactory dysfunction observed in preclinical AD.

**FIGURE 2:**
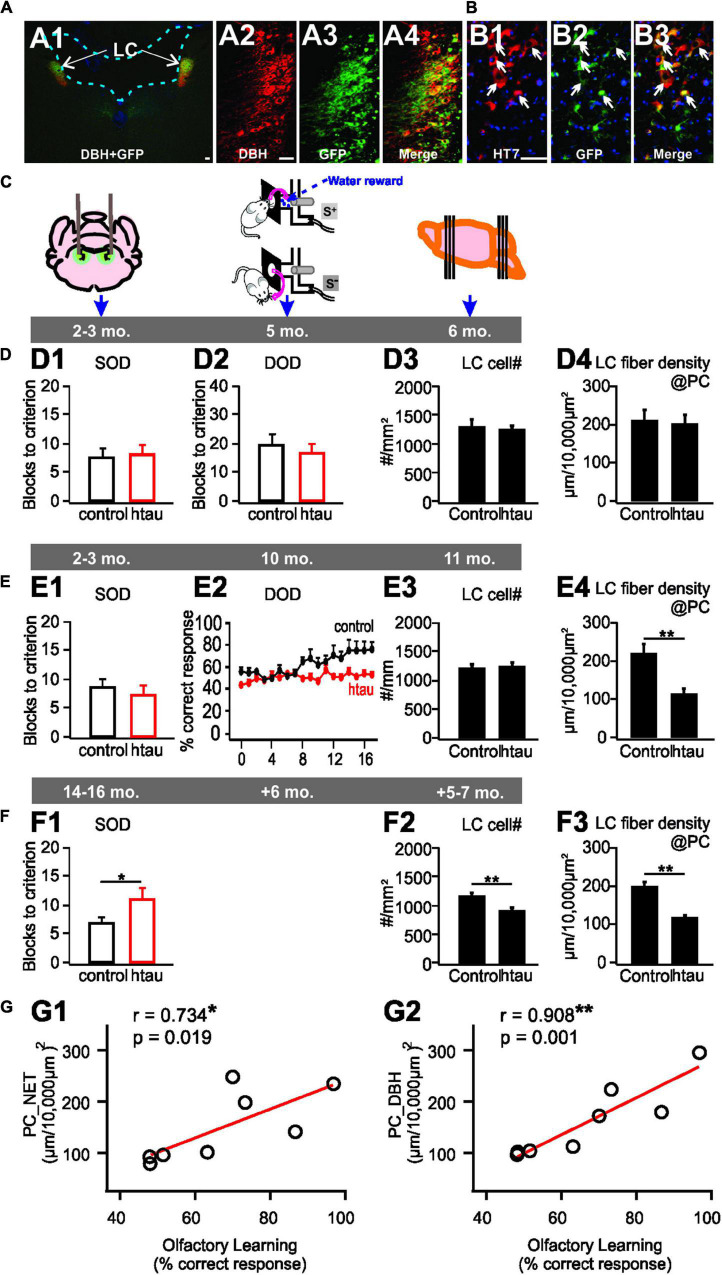
A locus coeruleus pretangle (LC) tau model in rats. **(A1–A4)** htauE14-GFP uptake in the LC co-localizes with DBH + LC neurons. **(B1–B3)** GFP co-localizes with HT7 indexing human tau. **(C)** Schematics of the timeline of AAV infusion, odor discrimination training, and histology. **(D1–D4)** No odor discrimination deficiency **(D1,D2)**, or LC cell loss **(D3)**, or LC fiber degeneration **(D4)** in young 6 month-old rats infused with htauE14 at 2–3 month-old. **(E1–E4)** Impairment in similar (SOD), but not dissimilar odor discrimination (DOD) learning in 10 month-old rats infused with htauE14 at 2–3 month-old **(E1,E2)**, no LC cell loss **(E3)** but LC fiber degeneration in the piriform cortex (PC) was observed **(E4)**. **(F1–F3)** Deficiency in SOD **(F1)**, LC cell number loss **(F2)** and fiber degeneration **(F3)** in 17–20 month-old rats infused with, htauE14 at 12–14 month-old. **(G1,G2)** LC fiber density in the PC is correlated with olfactory discrimination learning performance. NET, norepinephrine transport; DBH, dopamine beta-hydroxylase; HT7, Human Tau 7. Scale bars, 50 μm. Adapted and modified from Ghosh et al. (2019) and Omoluabi et al. (2021).

In a recent pretangle tau model in rats (Figure 2), a human tau gene pseudophosphorylated on 14 sites (htauE14) was seeded in the LC to mimic the persistent phosphorylation of pretangles (Ghosh et al., 2019; Figures 2A,B). As in human tissue, pretangle tau in this model appeared in the somatodendritic compartment of the LC and spread from LC to other neuromodulatory groups such as serotonergic dorsal raphe neurons. LC neurons bearing this pretangle tau underwent degeneration with prolonged time. When htauE14 is infused at 2–3 months old (mimicking early onset of human pretangle tau (Braak et al., 2011), LC cell counts and NE fiber density in the PC, 3 months after htauE14 seeding, did not differ from control brains. Functionally, the acquisition of a difficult olfactory discrimination, which requires LC input to PC (Shakhawat et al., 2015), was not impaired (Figures 2C,D). However, at 7–8 months post-htauE14 infusion, NE fiber density in the PC decreased and difficult odor discrimination was impaired (Figure 2E). However, expression of the NE transporter, NET, was also reduced and was concomitant with β_1_-adrenoceptor up-regulation in the PC. The NE levels in the PC are yet to be determined. When htauE14 is seeded at middle-age (12–14 months old), the pretangle tau pathology progressed faster. At 5–6 months post-infusion, LC cells started to reduce, correlating with a deficiency in a simpler version of the odor learning (Figure 2F). Odor discrimination and identification in humans is sensitive to brain aging (Doty et al., 1984) and may relate to declining NE fiber densities. This animal model provides the first evidence that LC pretangle tau pathology associated with PC adrenergic dysregulation may drive olfactory dysfunction in preclinical AD stages. The causal effects of PC adrenergic support in olfactory dysfunction observed in this model is further demonstrated by a more recent study (Omoluabi et al., 2021). Combining the pre-tangle tau model with optogenetic stimulation, we demonstrated that a 6 week chronic, learning- and positive valence-promoting LC phasic patterned activation (Ghosh et al., 2021), prevented LC fiber degeneration in the PC, and restored olfactory discrimination learning. Additionally, the adrenergic fiber density in the PC is positively correlated with odor discrimination performance (Figure 2G). Thus, the pretangle tau rat model suggests pre-clinical olfactory deficiency is strongly correlated with adrenergic deficiency in the PC. Non-invasive techniques, such as PET imaging with a radioligand for the NE-transporter (Arakawa et al., 2008), may be useful in testing this relationship in a human clinical setting.

Noradrenergic alteration and parallel olfactory dysfunction have been demonstrated in other transgenic rodent models of AD. Tg2676 mice exhibit neurodegeneration in LC along with olfactory deficits (Guerin et al., 2009), while TgCRND8 mice show depressive behavior and impaired object recognition memory that are correlated with low NE at the tissue level (Francis et al., 2012). Triple transgenic (3xTg-AD) mice also display reduced olfactory memory performance and AD pathology in these mice is exacerbated by β2 adrenergic receptor antagonists, suggesting altered noradrenergic signaling (Cassano et al., 2011; Branca et al., 2014). Double transgenic APP/PS1 mice with dopamine beta-hydroxylase (DBH) knockout inhibits NE synthesis and exacerbates hippocampal LTP and spatial memory deficiency observed in either APP/PS1 or DBH knockout alone (Hammerschmidt et al., 2013). These transgenic mice also display specific vulnerability to neurodegeneration within olfactory networks (Saiz-Sanchez et al., 2012). LC lesion with *N*-(2-Chloroethy)-*N*-ethyl-bromo-benzylamine (DSP4) also weakened olfactory discrimination ability and short-term memory in the APP/PS1 model (Rey et al., 2012). Impressively, behavioral deficiencies can be ameliorated by enhancing NE in several of these models. α_2_-adrenoreceptor antagonists administered in drinking water or osmotic minipumps prevented age-related spatial memory deficits in APP/PS1 mice (Scullion et al., 2011) and improved object recognition in TgCRND8 mice (Francis et al., 2012). Spatial memory deficits in APP/PS1 mice with DBH deficiency were also improved by subcutaneous injections of the NE precursor L-threo-dihydroxyphenylserine (Hammerschmidt et al., 2013).

## Norepinephrine Modulation of Calcium Channels in Alzheimer’s Disease: A Hypothesis

**FIGURE 3:**
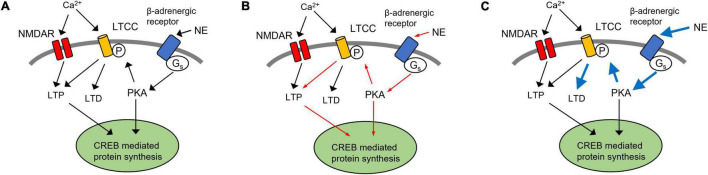
A hypothetical model of LTCCs in NE dysregulation in Alzheimer’s disease. **(A)** LTCC contribute to both LTP and LTD in olfactory learning. In aged rats, LTD in the piriform cortex (PC) is LTCC-dependent (Rajani et al., 2021). Thus, inhibition of LTCCs in the aging piriform cortex blocks LTD and could enhance learning (Maziar et al., 2022). **(B)** Following the degeneration of noradrenergic inputs from the LC, a decrease in NE signaling could result in LTCC hypofunction, resulting in the reduction of LTCC-dependent LTP (red arrows). **(C)** Alternatively, a compensatory increase in extracellular NE and adrenergic receptor expression may lead to enhancement of LTCC-mediated LTD (blue arrows). LTP, long term potentiation; LTD, long term depression; PC, piriform cortex; NE, norepinephrine; LC, locus coeruleus; LTCC, L-type calcium channel; NMDAR, *N*-methyl-D-aspartate receptor.

Key components of NE-mediated enhancement of synaptic plasticity within the PC are the L-type calcium channels (LTCCs). LTCCs are downstream of β-adrenergic receptors, via a G_*s*_-mediated cAMP-PKA pathway to produce an increase in Ca^2+^ influx and CREB-mediated protein synthesis and the initiation of long-term memory formation (Ghosh et al., 2017a; Man et al., 2020). Recently, we demonstrated that similar to the hippocampus, there is an age-dependent increase in the contribution of LTCCs to LTD in the PC, concurrent with a decreased role for NMDARs (Rajani et al., 2021). Moreover, inhibition of LTCCs in the aging piriform cortex blocks LTD (Rajani et al., 2021) and could enhance learning (Maziar et al., 2022). The increased significance of LTCCs during aging may provide some insight into the susceptibility of the aged PC to AD-related changes of the LC-NE pathway (Figure 3). LTCCs contribute to both LTP and LTD in olfactory learning (Figure 3A). A decrease in NE input to the PC following LC degeneration may cause reduced activation of LTCCs, diminishing LTCC-mediated LTP and impairing protein synthesis-dependent long-term odor memory formation (Figure 3B). On the other hand, a compensatory increase in extracellular NE levels or adrenoceptors, which have been correlated with age-related cognitive decline and AD (Wang et al., 2013; Gannon and Wang, 2019), may cause LTCC hyperfunction, leading to enhanced LTD (Figure 3C). Such dysregulation has been suggested in models for age-related cognitive decline (Thibault et al., 2007) and may be exacerbated under the influence of higher NE signaling. The resulting impact of AD pathology on extracellular NE levels within the PC remains to be determined.

## Conclusion and Outlook

Neurodegeneration of the LC and dysregulated noradrenergic function correlate with AD progression and olfactory dysfunction during preclinical stages. Further exploration of cellular and molecular interactions within these regions may help to characterize preclinical symptoms during this critical window. Future perspectives will require an understanding of AD pathology on multi-regional (OB and PC) extracellular NE, noradrenergic receptor expression, and should involve the interaction of both Aβ and tau pathologies on aging individuals.

Preserving LC neuronal health and function could be key to prevent or reverse AD. Promoting phasic LC firing patterns and reducing stress-inducing high tonic activity appear to be beneficial in animal models (Omoluabi et al., 2021). Life exposures such as education, novel environment, cognitive tasks that are associated with LC phasic activity could enhance the neural reserve to delay or reduce AD-related pathology (Xu et al., 2020), whereas chronic stress that is associated with high tonic LC activity increases AD risk (Wilson et al., 2011). We propose that LC degeneration at pre-clinical stages drives early olfactory deficits. The availability of LC imaging methods (Betts et al., 2019) enables the examination of the relationship of LC integrity and olfactory function in living humans and could provide insight into progression of neurodegenerative diseases.

## Author Contributions

VR and QY contributed equally to the conceptualization, wrote the manuscript, and approved the submitted version.

## Conflict of Interest

The authors declare that the research was conducted in the absence of any commercial or financial relationships that could be construed as a potential conflict of interest.

## Publisher’s Note

All claims expressed in this article are solely those of the authors and do not necessarily represent those of their affiliated organizations, or those of the publisher, the editors and the reviewers. Any product that may be evaluated in this article, or claim that may be made by its manufacturer, is not guaranteed or endorsed by the publisher.
